# Linguistic Markers of Processing Trauma Experience in Women’s Written Narratives During Different Breast Cancer Phases: Implications for Clinical Interventions

**DOI:** 10.5964/ejop.v11i4.991

**Published:** 2015-11-27

**Authors:** Maria Luisa Martino, Raffaella Onorato, Maria Francesca Freda

**Affiliations:** a Department of Humanistic Studies, Federico II University, Naples, Italy; Aalborg University, Aalborg, Denmark

**Keywords:** linguistic markers, expressive writing, narrative, emotional and cognitive processing, breast cancer, clinical interventions

## Abstract

Research into the change processes underlying the benefits of expressive writing is still incomplete. To fill this gap, we investigated the linguistic markers of change in cognitive and emotional processing among women with breast cancer, highlighting the differences and peculiarities during different treatment phases. A total of 60 writings were collected from 20 women: 10 receiving chemotherapy and 10 receiving biological therapy. We performed a series of repeated measures ANOVA for the most meaningful LIWC linguistic categories, including positive/negative emotions and cognitive processes, to assess change over three sessions. Results demonstrated a significant increase in the positive emotions category for the entire group of women, with particular relevance for the biological therapy group of women, and a marginally significant (p = .07) greater use of words indicating cognitive processes for women receiving biological therapy. For the negative emotions category time was significant for the whole group of women, showing a peak of use in the second session of writing. Peculiar differences in the linguistic markers of processing trauma were observed between the two groups. Although the writing intervention is a support for both groups of women, it seems to be beneficial when there is a large time gap since the administration of chemotherapy and, thus, when the patient can revisit the experience. The relationship of the illness with life can be rearticulated, and the writing becomes a space for resignifying the traumatic cancer experience.

## Emotional and Cognitive Processing Through Expressive Writing During a Traumatic Breast Cancer Experience

The onset of an illness such as breast cancer is considered to be a traumatic experience for women who cope with long and arduous therapeutic treatment. Depending on the staging and treatable areas, from a medical viewpoint, the treatment includes local interventions such as surgery and radiotherapy or systemic interventions such as chemotherapy and hormonal therapy (Cordova et al., 2007). The difficulty in identifying a single stressful event, the internal source of the stressor factor, and the temporal continuity of the of the stressor experience are the core elements that characterize cancer as a traumatic experience unique and different from the others (Mehnert & Koch, 2007).

These treatments result in numerous side effects, such as fatigue, pain (Koopman, Hermanson, Diamond, Angell, & Spiegel, 1998), hair loss, and temporary and permanent changes in physical appearance, reduction in the quality of life, and difficulties with spouses/caregivers (Bonnaud-Antignac, Hardouin, Leger, Dravet, & Sebille, 2012). During the period of active treatment, the woman is faced with the difficult task of living with this “new” condition, which can strengthen or weaken the process of remodeling the patient’s self esteemand her ideal body (Fobair et al., 2006).

Breast cancer and its treatment in women cause a set of psychic disruptions that challenge femininity and provoke anxiety, depression, guilt feelings, isolation, worthlessness, distrust, and psychological distress. This traumatic event suddenly confronts the woman with a new type of information regarding the world; this information defies the person’s preexisting mental schemas and threatens one’s basic assumptions about the self and world, constructing a gap between appraised meaning and global beliefs, between global and situational meanings, between emotion and cognition that interrupt the continuity of life (Freda, De Luca Picione, & Martino, 2015; Janoff-Bulman, 2004).

The two principle treatment phases for breast cancer are chemotherapy and biological therapy that are administered at two different time points and are consequential for the therapeutic and pharmacological postoperative assessment, to which women with breast cancer are generally subjected for a very long time. The first treatment phase is the pharmacological mode that is administered in cycles. This destroys the cancer cells and is particularly invasive; however, it does not guarantee a certain prognosis of life. Biological therapy, the most promising phase in terms of prognosis, is a therapeutic approach with a reduced toxic effect that directly interferes with the functional expression of some genes, prevents the tumor from growing, and prevents a relapse because it coincides with a phase after the chemotherapy.

In this scenario, expressive writing interventions promote a beneficial meaning-making and integrative process of traumatic experience into one’s own life story, thereby constructing a narrative that connects emotion and cognition shattered by trauma and supporting health; physical benefits and emotion regulation (Boals, 2012; Boals, Banks, Hathaway, & Schuettler, 2011; de Campora, Giromini, Larciprete, Li Volsi, & Zavattini, 2014; Freda, De Luca Picione, & Martino, 2015; Martino, Freda, & Camera, 2013; Schutte, Searle, Meade, & Dark, 2012; Vrielynck, Philippot, & Rimé, 2010). The narrative is a space for transformation and resignifies the traumatic experience, through which the narrator reconstructs a broken self-narrative story after a traumatic experience (Angus & McLeod, 2004; Hermans, 2003; McAdams, 2008; Neimeyer, 2002).

The narration includes the role of a semiotic device, whereby the traumatic experience is reactualized in the here and now of the narrative setting. Through plot development during the narrative process, the writer sets up processes of semiotic connection that can promote change and knowledge, because he/she strives to find a configuration for events in the discourse that can make sense of the experience, even if temporarily, and thus promote integration of the trauma (Freda & Martino, 2015; Greenberg & Paivio, 2003; Margherita, Gargiulo, & Martino, 2015; Margherita, Martino, Recano, & Camera, 2014). Therefore, the narrative allows us to understand these changes and the subjective way by which people understand and connect with these transformations, observe how these transformations are constructed, and emerge in the stories of the subjects (Pals & McAdams, 2004).

On one hand, the evaluation of the benefits of the writing technique, in the context of breast or ovarian cancer, has been highlighted in the literature, showing a reduction of symptoms related to avoidance and approach to their emotions (Zakowski, Ramati, Morton, Johnson, & Flanigan, 2004) or a reduction of distress and symptoms related to the condition of evasion (Stanton et al., 2002). On the other hand, understanding of the change processes that underlie the beneficial effects of writing is incomplete (Freda & Martino, 2015; Stone, Smith, Kaell, & Hurewitz, 2000).

To achieve a better understanding of the processes of change activated by writing as they occur, there must be an analysis of words indicating cognitive and emotional processes, which are present within the written stories and are the types of mechanisms that mediate adaptation to the traumatic event (Pennebaker, Booth, & Francis, 2007).

Borrowing knowledge from linguistics, Pennebaker and colleagues (2010) have focused on the role of language. The words reflect but do not “cause” the states of mind. The words reflect changes in the way people think. Language, in this sense, is an epiphenomenon that offers information about who we are and simulateneously is a tool that provides a mechanism for effecting change (Pennebaker, Facchin, & Margola, 2010; Tausczik & Pennebaker, 2010). Therefore, what prompts the writingis a cognitive processing of the traumatic experience that becomes increasingly complex throughout the writing sessions and includes meaning making, insight, construction of a coherent narrative, integration of the experience into the patient’s own world view or change thereof, reinterpretation of the event as an opportunity for growth (Baikie & Wilhelm, 2005), and correction of emotional dysregulation that facilitates control over emotions and builds a new sense of mastery and efficacy (King, 2001).

In particular, in the context of cancer in women, few studies have explored the processes underlying the effects of writing; moreover, few studies have explored the linguistic markers and the way of narrative articulation of the cognitive and emotional processes in the writings of women with breast cancer. Most of the investigations have focused attention on correlational, predictive, or mediation studies between the health and physical benefits of writing and use of words. Previous research (Low, Stanton, & Danoff-Burg, 2006), conducted on the writings of women with breast cancer, revealed no association between the use of words and emotional and cognitive health benefits.

A study on Italian hospitalized patients (Iacono, Donati, & Solano, 2003) showed a correlation only between the use of emotional words (positive and negative) and health objective of subjects (days of hospitalization), with no significant difference between the use of cognitive and emotional words and the progress of discussions between patients with bladder papilloma with postoperative long or short hospital stay.

Several studies (Pennebaker, 2002; Pennebaker, Mehl, & Niederhoffer, 2003; Ramírez-Esparza & Pennebaker, 2006) have revealed that the greater use of positive emotional words during the writing sessions, as well as a gradual increase in the use of general cognitive words during the meetings, was associated with greater health benefits than an intermediate use of negative words. The use of emotional words proved to be a predictor of health benefits, particularly the progressive increase of cognitive words (cause and intuition) (Pennebaker, Booth, & Francis, 2007). Schwartz and Drotar (2004) demonstrated that a decrease in negative emotion together with an increase in cognitive processing facilitated by written emotional disclosure has beneficial effects on the physical health-related quality of life. The use of certain words, with particular reference to emotional and cognitive matter, reflects that the person is starting a process of building a coherent story, putting her thoughts and emotions into words, and making her way trying to find causes and make sense of the event, starting are reflexive and meta-reflexive process to promote health benefits (Pennebaker, Facchin, & Margola, 2010).

To enrich the comprehension of the processes underlying the benefits of expressive writing for women with breast cancer (Martino, Freda, & Camera, 2013), we investigated the linguistic markers of change by which the writing intervention reconstructed the patient’s narrative about the traumatic experience. We also assessed whether the writing intervention has supported the different ways of expressing their experiences in words and reorganizing the traumatic event (emotional and cognitive processing of the traumatic event) at different stages of treatment: chemotherapy and biological therapy. Despite the absence of specific reference literature on the subject of breast cancer, it was expected that we would be able to detect a gradual increase in the use of cognitive words and an increased use of words for positive emotions compared with a moderate use of words for negative emotions. Regarding the two phases of the illness, we meant to compare not only two different aspects of the therapeutic treatment but also two different moments of the traumatic experience: the phase of chemotherapy nearest to the traumatic acme of the communication of diagnosis and its uncertainty and the phase of biological therapy closer to a phase of life when the reconstruction and return to everyday routines take place. Such an understanding of therapeutic writing allows for the ability to reflect on the most benefit timing within which one can propose writing interventions and reflect about its functions.

## Method

### Participants

This study was conducted in two hospitals in southern Italy, both of which are national centers of reference for the treatment of oncological illnesses. The study was approved by the ethical committee of the hospitals. From the medical records, during the year 2013, 20 women were identified on the basis of the cancer stage and phase of the treatment (the participants were required to have non-metastatic breast cancer and be in the postoperative drug treatment phase) and asked to participate in our study. These women were categorized into two groups: 10 patients recieving chemotherapy and diagnosed in the preceding 6 months and 10 patients receiving biological therapy and diagnosed in the preceding 12 months. Participation in the study was voluntary after signing the informed consent; nobody refused to participate. The patients in the chemotherapy group were aged between 37 and 55 years (*M* = 45.3; *SD* = 10.6), and those in the biological therapy group were aged between 36 and 60 years (*M* = 49.1; *SD* = 7.78). The two groups were homogeneous for age, nationality, and socioeconomic status.

### Tools and Procedures

Within the greater panorama of expressive writing, we used the expressive writing technique (written emotional disclosure) as the model proposed earlier (Martino, Onorato, D’Oriano, & Freda, 2013; Pennebaker, Facchin, & Margola, 2010), which is used in the context of breast cancer traumatic experiences (Low, Stanton, & Danoff-Burg, 2006; Stanton et al., 2002).

For adapting the writing technique to the aim of this study, we asked the participants to write about the traumatic experience of breast cancer. The meetings occurred in an ad hoc room of the hospitals in conjunction with standard medical examinations. The writing sessions lasted 30 minutes each and were structured over time with an interval of 21 days to promote, between one session and the other, the in-depth progression of emotional and cognitive processing.

### Data Analysis

Initially, for analyzing the written texts, we used the Linguistic Inquiry and Word Count (LIWC2007), a software for text analysis based on the number of words belonging to 80 linguistic categories (Freda, Esposito, & Quaranta, 2015; Freitag, Grimm, & Schmidt, 2011; Pennebaker et al., 2007). The corpus of analysis consisted of 60 transcribed texts (3/subject). The texts were analyzed after integration of the Italian dictionary to implement the number of recognized words reaching an average of 80% of recognized words.

The LIWC2007 consists of > 4,500 terms that are included by numeral recognition in one or more categories simultaneously. It involves classifications that are structured into categories and subcategories; for example, macrocategory psychological processes is included in the cognitive processes category, which, in turn, contains causality and insights in its interior subcategories and so on for each dimension. Its internal structure consists of two basic components: a program that calculates the percentage of words contained in the various linguistic categories, creating an output file, and a language dictionary.

The output produced by the analysis includes as many as 32 categories indicating psychological constructs and as many categories indicating paralinguistic dimensions, personal interests, and punctuation. In the present work, we particularly focused only on certain linguistic categories that are considered in the literature to be the most central, referring to emotional and cognitive processing, in terms of positive emotions (i.e., good, love, happy), negative emotions (i.e., worthlessness, ineffectiveness, hatred, enemy) and cognitive processes (i.e., I recognize, I know, in fact, why, then, I think, consider) (Hoyt & Pasupathi, 2008; Moore & Brody, 2009; North, Meyerson, Brown, & Holahan, 2013).

Subsequently, we performed a series of repeated measures analysis of variance (ANOVA), with a two-way mixed factorial design, to verify the best hypothesis proposals. In all cases, a 5% maximum threshold error was assumed.

For each preselected language category (positive/negative emotions and cognitive process), the dependent variable was defined as the score resulting in the LIWC analysis for each writing session (T1-T2-T3) and as a factor between the groups and the treatment-therapeutic phase that the women were undergoing (chemotherapy and biological therapy).

## Results

Computer analysis of texts was performed to control the interaction between linguistic categories and therapy.

Linguistic categories and time were also analyzed to extrapolate mean scores, which was calculated as the progress of the three writing sessions. In addition, scores were determined for the categories of positive emotions, negative emotions, and cognitive processes for the groups in biological therapy, for the groups in chemotherapy, and for the groups as a whole (Table 1).

**Table 1:** Descriptive Statistics of the Scores of Linguistic Categories (Positive Emotions, Negative Emotions, Cognitive Processes), Reported From Three Writing Sessions (T1-T2-T3) for Biological Therapy, Chemotherapy, and Entire Group

Group	Positive Emotions						Negative Emotions						Cognitive Processes					
	T1		T2		T3		T1		T2		T3		T1		T2		T3	
	*M*	*SD*	*M*	*SD*	*M*	*SD*	*M*	*SD*	*M*	*SD*	*M*	*SD*	*M*	*SD*	*M*	*SD*	*M*	*SD*
Chemotherapy *n* = 10	7.60	5.04	12.88	5.25	12.45	5.76	3.52	2.04	7.72	2.17	8.09	4.76	7.71	3.27	9.20	2.69	8.21	2.87
Biological Therapy *n* = 10	11.90	5.22	15.20	3.74	17.00	4.40	5.98	3.63	8.63	2.96	6.82	2.35	9.07	4.08	9.42	2.47	11.47	4.46
Entire group *N* = 20	9.75	5.46	14.04	4.59	14.73	5.51	4.75	3.13	8.18	2.57	7.45	3.71	8.39	3.67	9.31	2.52	9.84	4.02

**Table 2:** Repeated Measures ANOVA: Main Effects Between Groups, Related to the Linguistic Categories (Positive Emotions, Negative Emotions, Cognitive Processes) for Therapy and Time

Factor	Positive Emotions		Negative Emotions		Cognitive Processes	
	*df*	*F*	*df*	*F*	*df*	*F*
Therapy	1	6.5360	1	0.5270	1	3.7510
Time	1	10.234	1	7.389	1	1.321

The Repeated Measures Anova show the following main effects (Table 2). For positive emotions the main effects for therapy and time between the groups were significant, *F*(1, 18) = 6.53, *p* = .02, and *F*(1, 18) = 10.234, *p* = .005, respectively. These data show a greater use of positive emotions by women receiving biological therapy and a progressive increase of words indicating positive emotions in the whole group, over the course of the three writing sessions. Over the course of the three writing sessions, the biological therapy group showed a considerable increase between T1 and T3 (11.90 and 17.00, respectively) contrary to the chemotherapy group (7.60 and 12.45, respectively).

Regarding the negative emotions category, the factor therapy was not significant, *F*(1, 18) = 0.52, *p* = .48, while time was significant, *F*(1, 18) = 7.389, *p* = .014, showing a peak in the second session of writing (Table 1). In observational terms, we can read an interesting dynamic: a steady increase in the use of negative emotion words in the group receiving chemotherapy (the means were 3.52, 7.72, and 8.09, respectively, for T1, T2, and T3) compared with the biological therapy group, which showed an increasing trend at T2 and decreased again at T3 (5.98, 8.63, and 6.82, respectively, for T1, T2, and T3).

For the cognitive processes category the factor therapy approached significance, *F*(1,18) = 3.75, *p* = .07, thus suggesting a greater use of cognitive words for women receiving biological therapy. Observing the mean scores we note a mean increase between T1 and T3 in both groups, although the increase was noticeably greater in the biological therapy group (9.07 and 11.47, respectively). However, from a statistical point of view the factor time was not significant, *F*(1, 18) = 1.321, *p* = .27.

In summary, the results reveal that the entire group of women, regardless of the therapeutic phase they were going through, showed a statistically significant increase in the mean number of words indicating positive emotions between T1 and T3 (9.75, and 14.73, repectively), compared to words indicating negative emotions that tend to maintain a stable trend, while the mean scores for the words indicating cognitive processes, although not significant, increased gradually between T1 and T3 (8.39, and 9.84, respectively).

The analysis did not reveal any significant effects of the interaction time versus therapy for the three linguistic categories and, therefore, does not provide evidence in this regard.

## Discussion

Our results only partially confirmed our hypothesis. As hypothesized, we detected a statistically significant increase over the course of the three writing sessions for the linguistic category of positive emotions and negative emotions within the entire group of women; however, cognitive processes do not show statistically significant changes. Analysis of the effects between the groups (Table 2) confirms the statistically significant differences for the factor therapy, i.e., between the groups receiving biological therapy and chemotherapy, only in the scores for the categories of positive emotions. Looking at the trend of the mean scores for the various linguistic categories in question (Table 1), we can say that the level of positive emotions during the first writing session is more elevated for women receiving biological therapy than for those receiving chemotherapy. Given this initial difference, the group receiving biological therapy demonstrated a progressive increase in the use of words indicating positive emotions, with a marked increase during the third and final session of writing. This could possibly be interpreted by observing a connection with the patient’s therapeutic phase, a stage when concerns about the possible effects of therapy are attenuated; the body does not suffer with this type of therapy, as with the physical upheavals that accompany chemotherapy.

We interpret that the ability to use a greater number of words indicating positive emotions peculiar of women in biological therapy, which increases over time, could confirm the possibility to look beyond the cancer experience. This ability allows to look with a positive involvement to the present and future, integrating the event into one’s own personal lifestory, adopting positive resources and an increasing the use of optimistic strategies for trauma resolution. We believe that this is facilitated by the possibility to construct a dialogue, over the time course of the illness, with one’s own life, thanks to a view of the future that is, gradually, more attainable (Freda, De Luca Picione, & Martino, 2015; Freda & Martino, 2015).

Regarding the cognitive processes category, a statistically significant difference between the two groups of women was not observed. Starting from this assumption, observing the mean trend (Table 1) we note a peculiarity: the biological therapy group used cognitive words, particularly during the third and last session of writing. These data confirm the presence of a process of construction of a narrative meta-reflection, re-interpretation, and integration of the experience. We believe that the increased use of cognitive words is observable for women receiving biological therapy because of the presence of a greater distance from the traumatic acme of communication of diagnosis and the beginning of the arduous therapeutic treatment, as well as the inevitable gradual health improvements and the greater freedom from fear of death in the future. This results, in the latter stage, in an experience that is less threatening and, thus, able to be contemplated through distance, by adopting a meta-reflective lens aimed at constructing meaning and comprehension. A relationship with the future that is still grappling with the significance of the current time makes this process much more arduous for women receiving chemotherapy.

However, regarding the differences within the groups, there was no significant interaction between the different types of therapy, chemotherapy and biological, and the use of certain linguistic categories or the progression thereof in the three writing sessions. Maintaining an awareness of the differences among the subjects and the research design, this result is consistent with the results of the study by Iacono, Donati, and Solano (2003), which did not show a significant difference between the use of words and the cognitive and emotional advance of the meetings between subjects suffering from bladder papilloma, with long or short postoperative hospital stay. We found that women receiving biological therapy more often reference negative emotions in the second session, as a process of progressive connection between the events and emotions, including the negative ones that have characterized its traumatic story, negative emotions that the women receiving biological therapy can now label and feel. Women receiving biological therapies relive the traumatic experience and the painful onset of the illness and its therapeutic procedure by creating a process of identification (emotional labeling), acceptance of pain, and awareness of a connection between the events and the emotions, building meaning that allows them to be able to re-harmonize with the traumatic experience and future (Freda, De Luca Picione, & Martino, 2015).

As we had mentioned earlier, the entire group of women, regardless of the type of ongoing therapy, showed an increase of positive and negative emotion words, indicating the progress of the writing sessions. In this sense, it can be said that the intervention of writing is an activator of emotionally ideational processing of the present and future and their own projects within a narrative construction, which seems to have formed a connection between negative and positive; good and bad non-verbal aspects, still raw and not symbolized, and a search for expressive words (Bucci, 1997; Iacono, Donati, & Solano, 2003).

In any case, the lack of a significant interaction between the different types of therapy and the use of certain linguistic categories during the progress of the three sessions seems to confirm a beneficial use of the tool of writing, which has allowed the promotion of transformation processes and the connection of events, starting with the construction of a border and a frame, both internal and external, within which to share and preserve their story. We believe that this has supported or is supporting a process of integration experience (Martino, Onorato, D’Oriano, & Freda, 2013).

### Conclusions and Implications for Clinical Interventions

The intervention of expressive writing for women with breast cancer is an opportunity for re-transcription of the traumatic experience that led to an increase in the reorganization of the emotional trauma caused by the illness. Despite the limitations of this study related to the number of participants, the analysis made it possible, however, to throw light on the processes underlying the writing, the way of putting the traumatic event of illness into words, and the use and choice of cognitive and emotional linguistic markers to be able to relate and build integration. In particular, this study has allowed us to observe peculiar modes/ways that characterize the process of expressing in words and the construction of the meaning of personal experience depending on the therapeutic phase. We believe that these differences in go-through and transformation of the meaning of experience have played a mediating role within the specific relationship between the person, illness, and reality.

On one hand, the writings of women undergoing chemotherapy go through their experience focusing on the current time of the illness process and its therapeutic aspect that makes it more difficult to build a reflective process on past illness experience. In this sense, the future comprises the time of the continuity of the illness that is full of uncertainty and fear (Freda & Martino, 2015). On the other hand, women undergoing biological therapy reorganize their experience of illness, putting into words and re-experiencing itsmost painful aspects, but simulataneously being able to build harmonization in dialogue with the illness, present, and future. The construction of a frame of sense, with respect to the complexity (integration of emotion and cognition) of their experience, seems to proceed simultaneously with the ability to positively symbolize their future, being in a promising therapeutic phase.

Although the writing was a beneficial tool for all women, with a different degree and modality, it seems important to choose the type of psychological intervention in line with the specific phase of treatment and its cognitive–emotional processing style. It is also important to incorporate this kind of intervention within the psychological service of the hospital to provide these women the possibility to continue their meetings with a psychologist because the writing intervention is configurated, also, as an activator of psychological demand (Martino, Freda, & Camera, 2013; van Middendorp & Geenen, 2008). In addition, the context in which the expressive writing takes place must be considered as it can influence the use of language and the choice of linguistic markers by the participants (Corter & Petrie, 2008).

As implication of our study we believe that the possibility to reflect and meta-reflect about the illness experience and construct a coherent story is more possible when the expressive writing is proposed at a phase sufficiently distant from the traumatic climax of the illness and its arduous therapeutic process (chemotherapy). We believe that expressive writing could be more useful for women during a time in which they begin to understand the past experience of cancer and are able to construct a complex and meta-perspective framework for the events. This seems possible because the fear of death is less acute and the bodily conditions are promising for a positive future.

In conclusion, this preliminary research study holds implications for clinical work as well as for planning interventions to improve well-being in the oncology setting. Understanding the different ways in more detail and the path to narrative re-construction of the traumatic experience of breast cancer among women undergoing the specific therapeutic phase, as well as the distance from the communication of diagnosis, may help psychologists to propose an expressive writing intervention within a proper distance. It may help support a process of sharing and narrate the traumatic illness experience, respecting the patient’s internal and external lived times of experience. This refers to the ability to promote the choice of the best type of psychological support within a hospital setting, according to the phases of medical treatment as well as the distance from the traumatic acme of communication of diagnosis.

In our future research, we plan to increase the number of participants and compare other types and phases of oncological illnesses.
